# Health literacy, attitudes and preventive practices concerning mosquitoes and mosquito-borne infections – A questionnaire survey in a German community

**DOI:** 10.1016/j.onehlt.2025.101163

**Published:** 2025-08-22

**Authors:** Lukas Eicher, Andrea Weber, Julia Tobias, Andrea Verbitskii, Michael Leitzmann, Benedikt M.J. Lampl

**Affiliations:** a Department of Epidemiology and Preventive Medicine, University of Regensburg, Regensburg, Germany; b Division of Infection Control and Prevention, Regensburg Department of Public Health, Regensburg, Germany

**Keywords:** Mosquito-borne infectious diseases, Knowledge, Attitudes, Preventive measures, Protection from mosquitoes, Pre-travel health advice

## Abstract

**Introduction:**

Climate variability and non-environmental factors such as travel and migration pose an increasing risk of vector-borne infectious diseases to extratropical regions. The European Centre for Disease Prevention and Control has reported autochthonous transmissions of dengue or West Nile virus in Italy, France, Spain, and Germany. Raising awareness and implementing protective measures against mosquitoes will therefore become increasingly relevant in Germany in the future.

**Materials and methods:**

An observational cross-sectional study was performed between April 1 and July 31, 2024, deploying a paper-based anonymous questionnaire distributed to residents of Regensburg. The questionnaire included 19 questions covering demographic data, travel experience, knowledge about mosquitoes and protective measures, and attitudes and practices towards mosquito protection. Data were analyzed descriptively, and an ordinal logistic regression analysis was performed.

**Results:**

Most respondents showed basic knowledge about mosquito species, while awareness of breeding sites and vector-borne diseases was lower. Climate change was regarded as a relevant health concern by 89 % of participants; however, only 33.3 % perceived a current risk of mosquito-borne infections in Germany. More than half of the participants stated already protecting themselves from mosquitoes, and one third indicated they actively removed breeding sites from their surroundings. Pre-travel health advice, including mosquito-related information, had a positive impact on knowledge, attitudes and preventive practices.

**Conclusion:**

Awareness of the health risks associated with vectors, as well as specific knowledge about breeding sites, species, and mosquito-borne diseases, varied among participants. Our findings underline the educational potential in this area: specific aspects of vector-borne infections should be targeted to strengthen population health literacy in the future, for example, through focused information campaigns.

## 1 Introduction

Until recent years, mosquito-borne infections in central Europe had been perceived as a health threat limited mainly to tropical countries. Yet, at least until the 19th century, malaria was widespread throughout Europe, even in northern countries, and was eradicated only in the 1970s [[1], [2], [3]]. Different factors contributed to this eradication, such as drainage of swamps, use of insecticides, urbanization, environmental engineering, improvement of general health care and specific drug therapy, as well as economic advancement [3]. Nonetheless, potential vectors for malaria (genus *Anopheles*) remain prevalent in Europe [4].

Due to anthropogenic climatic change or climate variability, conditions for several vectors potentially transmitting viral or other pathogens have become suitable in central Europe. The Asian tiger mosquito (*Aedes albopictus*) occurs in regions north of the Alps. In Germany, for example, this is especially the case in the Upper Rhine valley (Baden-Württemberg, Rhineland-Palatinate, Hesse), however, *A. albopictus* has established isolated populations in Bavaria, Thuringia, and Berlin as well. On the other hand, a worldwide steep increase in reported dengue virus infections and imported cases in Germany was observed in 2024 [5]. As a consequence, taking into account migration and travel activities, the risk of autochthonous transmission generally increases, as competent vectors might be able to acquire pathogens from viremic/parasitemic individuals [6]. Accordingly, in recent years, the European Centre for Disease Prevention and Control (ECDC) reported autochthonous transmissions of dengue virus in Croatia, Italy, France, and Spain [7]. Yet, autochthonous transmission of dengue virus has not yet been recorded for Germany. This does not apply to the West Nile virus, which, most likely, was introduced to Europe by migratory birds and is transmitted primarily by species of the *Culex* genus [8]. Cases had initially been reported in southern European countries and, in recent years, also in parts of Germany [9]. Based on these observations, we hypothesize that vector-borne infections are emerging, or re-emerging diseases, respectively, and might be an increasingly common phenomenon in central Europe in the near future and, thus, can no longer be regarded as a health issue of (sub-)tropical regions, but rather, as a global health problem [6].

Informing the public and implementing protective measures against mosquitoes as potential vectors for infectious diseases will therefore be increasingly relevant in Germany. To date, only a limited amount of data exists on the knowledge, attitudes, and willingness to act regarding mosquito-borne infections and protection from mosquitoes on an individual level in the German population [4,10]. With the **Health Literacy on Mosquito-borne infectious diseases and Protection from Mosquitoes study** (Gesundheitskompetenz in puncto stechmückenübertragener Infektionen und Mückenschutz, GeKoMü-Studie), initial data on the above-mentioned aspects were collected in a cross-sectional survey of a regional sample from Germany. The objective of the project was to gain first insights into basic aspects in order to inform future research, measures, and campaigns.

## 2 Methods

### 2.1 Study design, period, population, and sample

This was an observational cross-sectional study based on a questionnaire provided at the Regensburg Public Health Department during a four-month time period between April 1, and July 31, 2024. The study sample was a convenience sample, comprised 598 adults (≥ 18 years of age), and was recruited from the population of the city and county of Regensburg (approximately 380,000 residents, corresponding to the local jurisdiction of the Regensburg Public Health Department). The study was announced at the administrative district office (Landratsamt Regensburg) and potential participants were actively approached during the study period. Participants were either visitors to the Public Health Department or employees of the district office not professionally involved in mosquito prevention, infection control, or related activities. If consent was given, a questionnaire was handed out. Participation in the survey was voluntary and anonymous. The project was submitted to the Ethics Committee of the University of Regensburg, which waived the requirement for ethical approval due to the nature of the study.

### 2.2 Questionnaire and data collection

The paper-based questionnaire included 19 items (statements or questions) covering three domains (knowledge, attitudes, and practices, KAP). Data were collected on demographics, travel experience, knowledge about mosquitoes, infections, and personal protective measures, attitudes towards mosquito protection, and participants' willingness to take action (**Suppl. Fig. S1**). Questions were based on previously conducted studies on KAP in other geographic regions [4,11,12]. For validation, the questionnaire was assessed for relevance and comprehensibility and pretested by medical staff of the Regensburg Public Health Department (*n* = 14), who were not involved as investigators.

The questionnaires were numbered consecutively. The data were transferred individually to an Excel spreadsheet by the principal investigator (LE) and randomly cross-checked by another co-author (JT) in terms of data completeness and correctness in 10 % of the questionnaires. Questionnaires were included in our analysis only if participants answered the demographic and travel experience questions as well as at least one additional question. For questionnaires that met these inclusion criteria, we could only analyze those items in which at least one possible option was chosen. Individual items were excluded if more than one answer was chosen in single choice or Likert-scale questions, or if “None of the above” was selected together with another answer option. Therefore, relative frequencies are not always based on the full sample size of *n* = 598, but rather on the number of analyzable (answered) items, where applicable.

### 2.3 Statistics

For descriptive statistics, we present absolute and relative frequencies of categorical variables. For each question of the knowledge section (Questions 7–9, Suppl. Fig. S1), a knowledge score was calculated based on the number of correctly answered questions: We added the number of true answers and subtracted the number of incorrect answers. For each participant, we then totaled the scores for all 3 knowledge questions to receive an overall knowledge score ranging from 0 to 13 (highest possible score). We defined scores from 0 to 6 as low and scores from 7 to 13 as high. Answers to attitudes and practice questions were rated using a 5-point Likert scale (ranging from 1 as “fully agree” to 5 “fully disagree”). A descriptive subgroup analysis was performed using PivotTables.

Ordinal logistic regression using a proportional odds model was performed to evaluate whether age, gender, education level, travel experience, or knowledge (independent variables, binary transformed) were associated with attitudes and/or practices concerning mosquito-borne infections and their prevention (dependent variables). Additionally, knowledge served as a dependent variable to investigate if it was associated with age, gender, educational level, or travel experience. Regression analyses were adjusted for age, gender and education. The log odds were computed, and the values of the cumulative events for the log odds were transformed into *p*-values. The regression coefficient β indicated whether the selected category of the independent variable shifted the dependent variable up (positive coefficient) or down (negative coefficient) on an ordinal scale. Data management and statistical analyses were performed using Microsoft Excel (spreadsheet program version 16.94) and R (statistical software version 4.4.2). We defined α = 0.05 as the level of significance.

## 3 Results

### 3.1 Data completeness, demographic characteristics, and travel experience

Overall, 603 questionnaires were returned, and 598 questionnaires met the inclusion criteria. In 86.8 % of these questionnaires (*n* = 519) all items were completed. A random cross-check of 60 questionnaires revealed no transmission errors.

The majority of participants were female (*n* = 357, 59.7 %) and between 20 and 40 years of age (*n* = 401, 67.1 %; Table 1). Persons who had graduated from high school (“Allgemeine oder fachgebundene Hochschulreife / Abitur”, see **Suppl. Fig. S1**) or university (*n* = 181, 30.3 %, or *n* = 286, 47.8 %) were overrepresented in the study sample. Only a small percentage of participants reported working in the medical or biological field (*n* = 20, 3.3 %). A major share of respondents was born in Germany (*n* = 523, 87.5 %). Of those born abroad, a majority originated from European (*n* = 34 of 74, 45.9 %) or Asian (n = 20 of 74, 27.0 %) countries. Approximately one third of subjects (*n* = 228, 38.1 %) reported having traveled to (sub-)tropical regions in the past, and a large share of those travelers reported having received pre-travel health advice (*n* = 180 of 228, 78.9 %). Most of these travelers reported having been advised on vector-borne diseases and protection from mosquito bites (*n* = 165 of 228, 72.4 %) before traveling. Almost all participants who had received pre-travel health advice stated that they had been provided with information about mosquito-specific aspects (n = 165 of 180, 91.7 %).

**Table 1:** Sociodemographic characteristics of study participants (*n* = 598).

Variables	Frequency (%)
Gender	
Women	357 (59.7)
Men	222 (37.1)
not stated	19 (3.2)
Age (years)	
< 20	49 (8.2)
20–40	401 (67.1)
> 40–60	107 (17.9)
> 60	29 (4.8)
not stated	12 (2.0)
Highest educational qualification	
No degree	16 (2.7)
Degree in the medical / biological field	20 (3.3)
Secondary school certificate	112 (18.7)
High school diploma	181 (30.3)
Professional training	82 (13.7)
College / university degree	286 (47.8)
Other qualification	7 (1.2)
not stated	2 (0.3)
Place of Birth	
Germany	523 (87.5)
Other	74 (12.4)
Europe	34 (5.7)
Asia	20 (3.3)
Africa	9 (1.5)
Americas	6 (1.0)
not stated	6 (1.0)
Travel experience	
Experienced in traveling to the (sub-)tropics	228 (38.1)
Received pre-travel medical advice	180 (30.1)

### 3.2 Knowledge about mosquitoes and protective measures

Respondents were requested to respond to questions regarding breeding sites, mosquito species, and potentially transmitted pathogens or diseases. The frequencies of specific answers are shown in Table 2. A total of 560 (93.6 %) participants identified stagnant water as a possible breeding ground for mosquitoes. “Open water sources” and “Artificial water sources” were identified as correct answers by 406 (67.9 %), and 262 respondents (43.8 %), respectively. Only a small proportion selected the incorrect answer “Dry soil” (*n* = 17, 2.8 %), but “Moist soil (flowerpots)” was chosen by half of the participants. None of the participants achieved the maximum score of 4 on this question. The highest score obtained was 3, reached by 76 participants (12.7 %). Most participants scored either 2 or 1 (*n* = 240, 40.1 %, or *n* = 210, 35.1 %, respectively).

**Table 2:** Knowledge about mosquitoes and protective measures (n = 598).

Variable	True (T) / false (F)	Frequency (%)
Where do mosquitoes prefer to breed? In…		
standing waters (lakes, ponds)	T	560 (93.6)
open water sources (rain barrels)	T	406 (67.9)
moist soil (flowerpots)	F	301 (50.3)
dry soil (farmland)	F	17 (2.8)
artificial water sources (plastic waste)	T	262 (43.8)
forests (on leaves)	NA⁎	106 (17.7)
None of these statements are correct.	F	3 (0.5)
Not analyzable		16 (2.7)
Which type of mosquito have you already heard of?		
Yellow fever mosquito	T	354 (59.2)
Malaria mosquito	T	522 (87.3)
Common house mosquito	T	415 (69.4)
I have never heard of any of these mosquito species.	F	24 (4.0)
Not analyzable		11 (1.8)
Which of the following diseases is transmitted by mosquitoes?		
Malaria	T	545 (91.1)
Dengue fever	T	377 (63.0)
Yellow fever	T	360 (60.2)
Zika virus	T	241 (40.3)
West Nile virus	T	142 (23.7)
Chikungunya virus	T	69 (11.5)
HIV	F	38 (6.4)
None of these diseases are transmitted by	F	11 (1.8)
mosquitoes.		13 (2.2)
Not analyzable		

⁎ This answer was excluded from further analysis due to ambiguity.

The Malaria mosquito was recognized by most participants (*n* = 522, 87.3 %), followed by the common house mosquito (*n* = 415, 69.4 %) and the yellow fever mosquito (*n* = 354, 59.2 %). A high share of respondents recognized all 3, or at least 2 of the mosquito genera (*n* = 248, 41.5 %, or *n* = 232, 38.8 %). A vast majority of participants (*n* = 545, 91.1 %) identified malaria as a potential disease transmitted by mosquitoes. Almost two thirds of respondents chose dengue fever and yellow fever as a correct answer. Zika virus, West Nile virus, and chikungunya virus were chosen by a considerably smaller proportion of respondents (*n* = 241, 40.3 %, *n* = 142, 23.7 %, and *n* = 69, 11.5 %). HIV was included as an incorrect answer, and was selected by only a small minority (*n* = 38, 6.4 %). The maximum possible score for this question was 6, achieved by 5.9 % of participants (*n* = 35).

We calculated an overall knowledge score (ranging from 0 to 13) by adding the scores of each participant's individual knowledge questions. The highest score recorded was 12 (*n* = 7, 1.2 %). Most respondents scored 5 to 8 points (*n* = 343, 57.4 %). Overall knowledge scores from 7 to 12 were more frequently achieved by participants who had traveled to (sub-) tropical destinations (*n* = 147 of 228, 64.5 % vs. *n* = 164 of 366, 44.8 %) as well as those who had received pre-travel health advice (*n* = 129 of 180, 71.7 % vs. *n* = 17 of 45, 37.8 %) including those who were more informed about mosquito-specific topics (*n* = 117 of 165, 70.9 % vs. *n* = 24 of 52, 46.2 %) compared to less informed participants. Overall scores from 6 to 12 were achieved more frequently by people with a college or university degree (*n* = 210 of 286, 73.4 % vs. *n* = 184 of 310, 59.4 %), male participants achieved scores from 8 to 12 more frequently (*n* = 93 of 222, 41.9 % vs. *n* = 109 of 357, 30.5 %). Knowledge scores showed no conspicuous tendency for different age groups.

### 3.3 Attitudes concerning mosquito protection

**Fig. 1:**
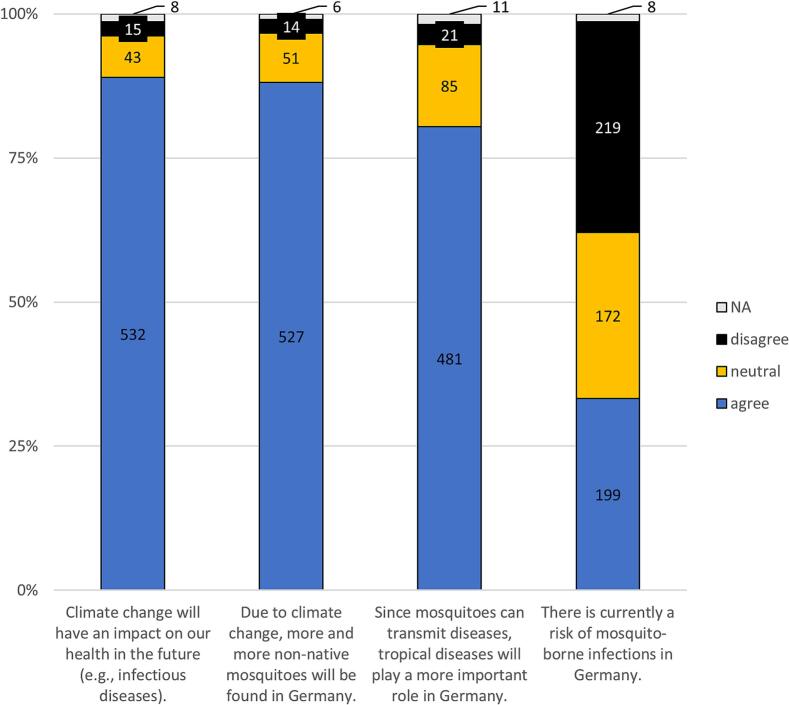
Opinions on potential health threats associated with mosquitoes (*n* = 598).

Most participants agreed that climate change in general would have an impact on their health in the future (*n* = 532, 89.0 %; Fig. 1). In this context, 88.1 % (*n* = 527) of participants believed that, due to climate change, non-endemic species of mosquitoes could permanently occur in Germany. Accordingly, a majority (*n* = 481, 80.4 %) regarded tropical diseases as more important in Germany in the future. On the other hand, 33.3 % (*n* = 199) felt there was currently a risk of mosquito-borne infections in Germany.

The majority of respondents believed that the government should regulate mosquito protection by law (*n* = 254, 42.5 %). When asked about innovative measures for protection from mosquitoes, such as genetic modification, answers were distributed almost equally: 209 participants (34.9 %) agreed, 170 (28.4 %) disagreed, and 201 (33.6 %) were neutral (not stated, *n* = 18, 3.0 %).

We investigated whether pre-travel health advice influenced the answers to the above-mentioned items. We compared participants who had received pre-travel health advice before traveling to the (sub-) tropics (*n* = 180) with those who had not (*n* = 45) and found a tendency towards more agreement with the following statements in the pre-travel health advice group: “Climate change will have an impact on our health in the future.”, *n* = 170, 94.4 % vs. *n* = 34, 75.6 %; “Due to climate change, more and more non-native mosquitoes will be found in Germany.”, *n* = 165, 91.7 % vs. *n* = 36, 80.0 %; “Since mosquitoes can transmit diseases, tropical diseases will play a more important role in Germany.”, *n* = 150, 83.3 % vs. n = 34, 75.6 %; “There is currently a risk of mosquito-borne infections in Germany.”, *n* = 64, 35.6 % vs. *n* = 11, 24.4 %.

### 3.4 Practices

**Fig. 2:**
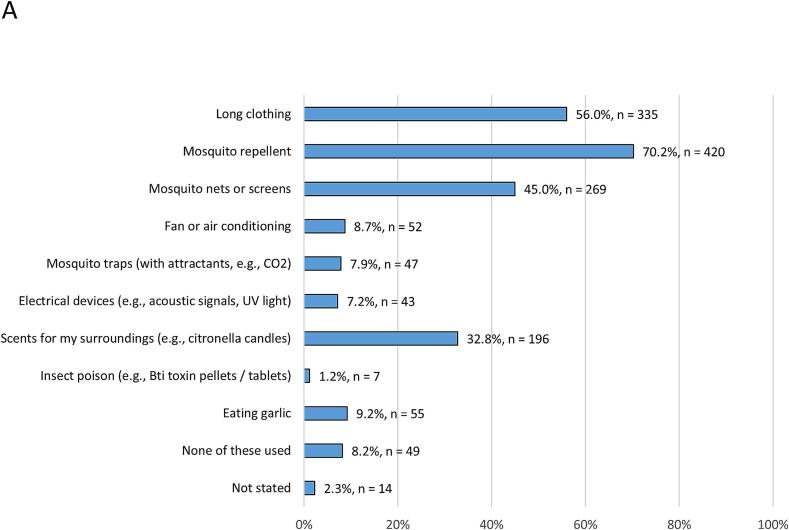

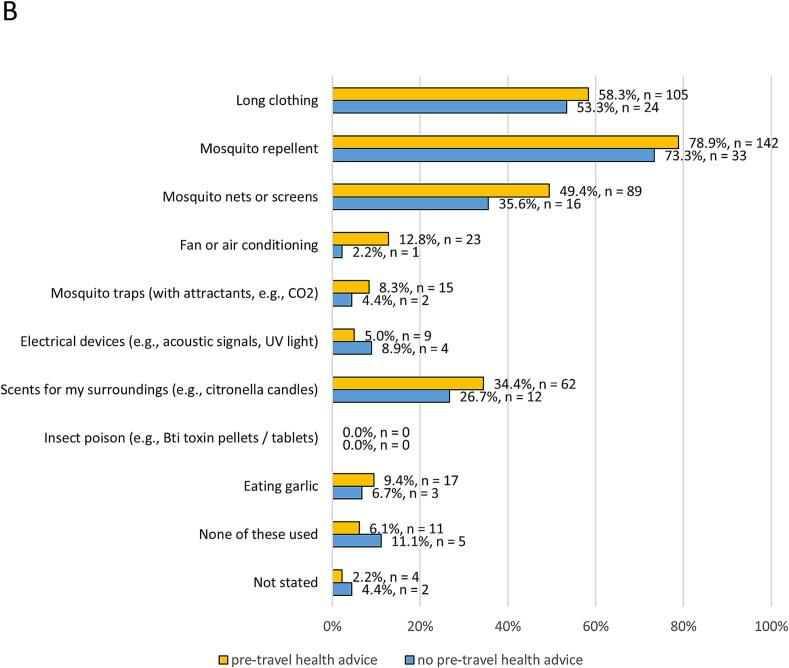

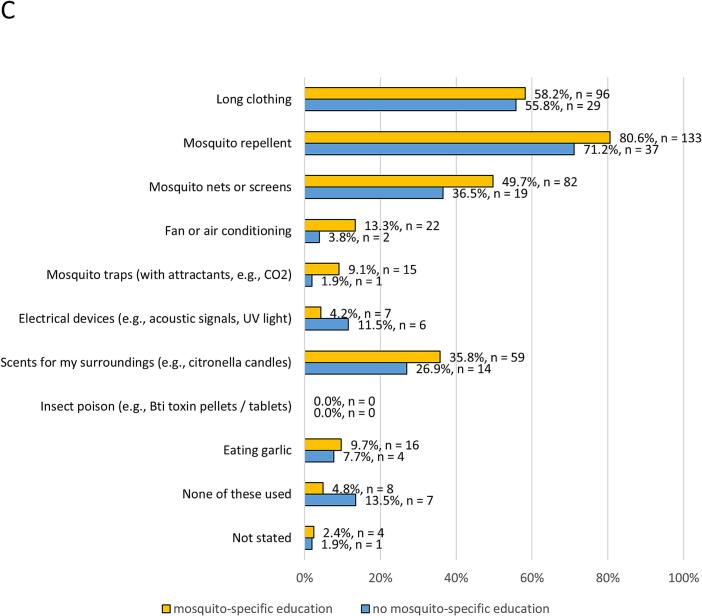
Protection measures used in everyday life. Relative frequencies relate to available data of each category. **A** Results for the total sample (n = 598). **B** Participants with (*n* = 180) and without (*n* = 45) pre-travel health advice. **C** Participants with (n = 165) and without (*n* = 52) specific education on mosquito-borne diseases and mosquito protection.

More than half of the participants stated they already protected themselves from mosquitoes (*n* = 339, 56.7 %). Participants were asked about their current practices concerning protection from mosquitoes (Fig. 2A). Use of repellents (*n* = 420, 70.2 %), long clothing (*n* = 335, 56.0 %), mosquito nets or screens (*n* = 269, 45.0 %), and scents for surroundings (*n* = 196, 32.8 %) were the most frequently stated response options. Overall, 223 persons (37.3 %) claimed they intentionally removed potential breeding sites for mosquitoes from their surroundings. Nearly all respondents stated a willingness to protect themselves from mosquitoes if they became a bigger health problem in the future (*n* = 539, 90.1 %).

When comparing protective measures between travel-experienced participants and those without such experience, no considerable differences were observed. However, pre-travel health advice was associated with an increased willingness to use protective measures (Fig. 2B). Moreover, participants who had explicitly been informed about mosquitoes and related risks stated using protective measures more frequently (Fig. 2C).

Compared to men, women more frequently reported consciously protecting themselves from mosquitoes (*n* = 219 of 357, 61.3 % vs. *n* = 113 of 222, 50.9 %; Table 3). The same held true for participants who had received pre-travel health advice (*n* = 119 of 180, 66.1 % vs. *n* = 22 of 45, 48.9 %) and those who were educated about mosquito-specific topics (*n* = 114 of 165, 69.1 % vs. *n* = 24 of 52, 46.2 %).

**Table 3:** Participants' willingness to engage in mosquito protection according to gender, age, travel experience, and knowledge score. Absolute and relative frequencies of agreement (“I strongly agree” and “I agree”) to statements 16, 18 and 19 of the questionnaire (see Supplemental Information, Fig. S1). Relative frequencies relate to available data of each category. P-values and regression coefficients (β) obtained from ordinal logistic regression analysis adjusted for age, gender and education.

	I already consciously protect myself from mosquitoes.	I consciously remove potential breeding sites for mosquitoes from my surroundings.	If mosquitoes become a bigger health problem in the future, I will consciously protect myself against them.
Gender⁎	*p* = 0.00244	*p* = 0.00272	*p* = 0.05978
	β = −0.4929	β = −0.4696	β = −0.3299
Female (n = 357)	219 (61.3)	146 (40.9)	321 (89.9)
Male (*n* = 222)	113 (50.9)	70 (31.5)	200 (90.1)
No statement (n = 19)	7 (36.8)	7 (36.8)	18 (94.7)
Age (years)⁎⁎	*p* = 0.000223	*p* = 0.000201	*p* = 0.216
	β = −0.7318	β = −0.6829	β = 0.2613
< 20 (*n* = 49)	21 (42.9)	10 (20.4)	42 (85.7)
20–40 (*n* = 401)	220 (54.9)	142 (35.4)	368 (91.8)
> 40–60 (*n* = 107)	73 (68.2)	53 (49.5)	95 (88.8)
> 60 (*n* = 29)	16 (55.2)	12 (41.4)	22 (75.9)
No statement (n = 12)	9 (75.0)	6 (50.0)	12 (100.0)
Travel experience⁎⁎⁎			
Experienced in traveling to the (sub-)tropics	*p* = 0.0984	*p* = 0.833	*p* = 0.225
	β = −0.2757	β = 0.03367	β = −0.20496
Yes (*n* = 228)	143 (62.7)	85 (37.3)	210 (92.1)
No (*n* = 366)	196 (53.6)	137 (37.4)	328 (89.6)
No statement (n = 4)	0 (0.0)	1 (25.0)	1 (25.0)
Received pre-travel health advice	*p* = 0.0138	*p* = 0.00111	*p* = 0.0800
	β = −0.8010	β = −1.04921	β = −0.6403
Yes (*n* = 180)	119 (66.1)	76 (42.2)	168 (93.3)
No (n = 45)	22 (48.9)	6 (13.3)	39 (86.7)
No statement (n = 3)	2 (66.7)	3 (100.0)	3 (100.0)
Received information about mosquito-borne diseases and mosquito protection	*p* = 0.00068	*p* = 0.07499	*p* = 0.068
	β = −1.0854	β = −0.5460	β = −0.6209
Yes (*n* = 165)	114 (69.1)	68 (41.2)	154 (93.3)
No (n = 52)	24 (46.2)	14 (26.9)	46 (88.5)
No statement (n = 11)	5 (45.5)	3 (27.3)	10 (90.9)
Results of overall knowledge score⁎⁎⁎	*p* = 0.03067	*p* = 0.004889	*p* = 0.06689
	β = 0.3583	β = 0.45293	β = 0.32986
High (*n* = 311)	195 (62.7)	128 (41.2)	289 (92.9)
Low (*n* = 260)	134 (51.5)	87 (33.5)	229 (88.1)
Not analyzable (*n* = 27)	10 (37.0)	8 (29.6)	21 (77.8)

⁎ Regression analysis based on binary gender (female or male).

⁎⁎ Regression analysis based on binary age groups (≤ 40 and > 40).

⁎⁎⁎ Regression analysis based on binary answers (yes or no, high or low).

More women than men claimed they actively removed potential breeding sites for insects from their surroundings (*n* = 146 of 357, 40.9 % vs. *n* = 70 of 222, 31.5 %). Pre-travel health advice (*n* = 76 of 180, 42.2 % vs. *n* = 6 of 45, 13.3 %) and information about mosquitoes and vector-borne diseases (*n* = 68 of 165, 41.2 % vs. n = 14 of 52, 26.9 %) was associated with increased willingness to implement this measure. Most respondents who achieved high total knowledge scores stated they took action against breeding sites in their environment (*n* = 128 of 311, 41.2 %).

Willingness to engage in protective measures in the future was high in both the high and low knowledge score groups (*n* = 289 of 311, 92.9 %, vs. *n* = 229 of 260, 88.1 %). Of note, this willingness was not strongly influenced by pre-travel health advice (*n* = 168 of 180, 93.3 % vs. *n* = 39 of 45, 86.7 %) or by specific education about mosquito protection (*n* = 154 of 165, 93.3 % vs. *n* = 46 of 52, 88.5 %).

### 3.5 Ordinal logistic regression analyses

Results of the ordinal logistic regression analysis are shown in **Suppl. Table S1** (Suppl. material). Travel experience, pre-travel health advice, specific education on mosquito protection, age above 40 years, high education level (university/college degree or higher; for all *p* < 0.001), and male gender (*p* < 0.05) was positively related to overall knowledge scores. Respondents who had received pre-travel health advice were more likely than others to agree with statements about the increasing spread of invasive mosquito species and the growing relevance of tropical diseases in Germany (for both p < 0.05). However, pre-travel health advice was not related to how respondents rated the impact of vector-borne diseases or climate change on their health, nor to their opinions on legislative measures or genetic modification (for all *p* > 0.05). Female gender, pre-travel health advice, a high overall knowledge score (for all *p* < 0.01), and age above 40 years were all related to increased willingness to take action against breeding sites in the environment (*p* < 0.001, Table 3).

## 4 Discussion

The present study of a regional convenience sample revealed that participants already possessed basic knowledge about mosquito species and their potential for transmitting infectious diseases. However, knowledge about breeding sites and vector-borne diseases was fragmentary. Many respondents were aware of the influence of climate change on health. On the other hand, a majority did not perceive a current risk of mosquito-borne infections in Germany. Around 40 % of participants felt that protection from mosquitoes should be a government responsibility, while there was no clear majority regarding the acceptance of genetic modifications of vectors as a preventive measure. We found that more than 50 % of participants stated that they already applied personal measures for protecting themselves from mosquitoes. Female gender and pre-travel health advice were positively correlated with participants' attitudes and practices against mosquitoes. Pre-travel health advice, including information on mosquito-relevant topics, also seemed to have an effect on personal knowledge and practices, underlining the need for and benefits of specific education on this topic.

In our study, more than 90 % of participants recognized stagnant water as a breeding site, but only 43.8 % chose artificial water sources as a correct answer. Studies in non-endemic regions reported similar results concerning knowledge about breeding sites [10,[13], [14], [15], [16], [17], [18]]. International studies emphasize that artificial breeding sites, such as plastic containers, are often not recognized as relevant [10,13,19]. Knowledge about mosquito species in our study was comparable to the moderate knowledge in non-endemic regions [10]. Vector-borne diseases, such as malaria, dengue, and yellow fever were identified by a rather large share of respondents, similar to other studies [18,19,25]. Zika virus, chikungunya virus, and West Nile virus were less known, which also resembled results from Italy, France, and Australia [13,14,20]. Generally, yet not surprisingly, a higher incidence in the region seems to be associated with better knowledge on the vector and the respective disease [10,13,14,21].

In our study, we found that travel experience, pre-travel health advice, and specific information about mosquitoes and protective behavior were associated with higher knowledge levels, consistent with previous literature [16,20,22]. Similarly, education and age were observed as influencing factors in several studies [15,21,23,24]. We found that the perception of health risks due to climatic change differed from other studies. A vast majority of our sample regarded climate change as relevant to health, and around 80 % believed tropical diseases were becoming increasingly important in Germany, contrasting with other studies from non-endemic countries reporting considerably lower awareness in this regard [10,13,20].

Genetic modification of mosquitoes found rather low acceptance (34.9 %), which could be explained by the lack of knowledge about biological control methods found in a study from France [13]. Yet, many respondents stated that they saw the government's responsibility in the issue, which is consistent with international findings [13,25,26].

In our study, 56.7 % of participants reported that they already protected themselves against mosquitoes. Results of other studies performed in non-endemic regions reported higher shares ranging from 60 to 90 % [13,16,27]. Women and respondents with higher awareness for the matter protected themselves more often, corresponding to results of other studies [15,22,28]. The share of participants in our survey stating that they actively removed breeding sites was below 40 %, contrasting with other studies [10,24,29]. Women and participants with higher levels of knowledge and higher health literacy generally seem more prone to take action [15,21,30]. 90.1 % of respondents in our survey would protect themselves in the future if mosquitoes became a greater problem. According to the review of Duval et al., this particular question was not asked in other studies from non-endemic countries [10].

Our findings emphasize the high potential for educational measures, provided that awareness for the topic can be raised. These measures should be applied in addition to situational prevention, such as reduction of breeding sites, measures of biological vector control, and legislation. Raising awareness about potential breeding grounds for mosquitoes is crucial for preventive measures to be applied more effectively [31]. Citizen science approaches, such as the Mücken-Atlas, which monitors mosquito species through public specimen submission in Germany, could help convey knowledge while promoting individual commitment.

While a number of studies concerning knowledge, attitudes, and practices regarding mosquitoes and mosquito-borne infections have been published internationally so far, this is, to our knowledge, the first study of its kind from a German population and provides first insights into key points that can be further addressed by future research and campaigns. This survey could be further validated and expanded to other public health departments to obtain more representative data on the wider German population. Due to the acquisition strategy of applying a paper-based questionnaire, we could reach a considerable number of participants within a rather short period of time. We cannot provide an exact participation rate. However, we estimate that more than every second to third person addressed was ready to participate, which would be a high response rate compared to usual figures. The topicality and local relevance of our research are underlined by the first confirmed finding of *Aedes albopictus* in the Regensburg area on Aug 7, 2024.

Our study has several limitations. First, this is an exploratory cross-sectional study that comes with the common limitations of an observational approach. Furthermore, our sample is rather small and comprises a convenience sample, and therefore, lacks representativeness. Women, people between the ages of 20 and 40, and respondents who graduated from high school or university are overrepresented. Another limitation is that we focused on climate change as a causative factor for vector-borne infections and did not specifically address other environmental and non-environmental factors in our questionnaire. Also, the present study is an explorative, mainly descriptive analysis, and sample size calculation was not performed because we lacked reliable data on prevalence or differences. Our questionnaire has not been validated extensively, so misinterpretation of individual questions by study participants might be an issue, resulting in potential information bias. Further, results might be prone to other types of bias due to the study design (e.g., selection bias). We cannot be certain whether our data reflect individual knowledge or whether questions were answered with external support, e.g., via smartphone and internet research. As our approach was exploratory, we did not adjust for multiple testing.

## 5 Conclusion

Our data indicate that persons of the surveyed sample were already aware of potential health risks associated with mosquitoes and possessed specific knowledge, e.g., about breeding sites, species, and diseases potentially transmitted by those vectors. However, knowledge and awareness still need to be further improved. Climate change is widely perceived as an influencing factor for health, but specific aspects of vector-borne infections must be addressed in more detail to strengthen the health literacy of the population in the future, e.g., through information campaigns. Our results need to be confirmed or refuted by future research on this topic.

## CRediT authorship contribution statement

**Lukas Eicher:** Writing – review & editing, Writing – original draft, Project administration, Methodology, Investigation, Formal analysis, Data curation, Conceptualization. **Andrea Weber:** Writing – review & editing, Resources, Methodology, Conceptualization. **Julia Tobias:** Writing – review & editing, Project administration, Data curation. **Andrea Verbitskii:** Writing – review & editing, Project administration. **Michael Leitzmann:** Writing – review & editing, Supervision. **Benedikt M.J. Lampl:** Writing – review & editing, Supervision, Methodology, Investigation, Conceptualization.

## Ethics

This is an observational study. The Ethics Committee of the University of Regensburg has confirmed that no ethical approval is required. The study was performed in accordance with the ethical standards as laid down in the 1964 Declaration of Helsinki.

## Funding

No funding was received for conducting this study.

## Declaration of competing interest

The authors declare that they have no known competing financial interests or personal relationships that could have appeared to influence the work reported in this paper.

## Data Availability

The data that support the findings of this study are available from the corresponding author upon reasonable request.

## Appendix A Supplementary data

Supplementary material: Original questionnaire (Fig. S1) and results of the ordinal logistic regression analyses (Table S1). Image 1
