# Supplementary material for: Health literacy, attitudes and preventive practices concerning mosquitoes and mosquito-borne infections – A questionnaire survey in a German community
Source: One Health. 2025 Aug 22;21:101163. doi: 10.1016/j.onehlt.2025.101163 (PMC12409310; doi:10.1016/j.onehlt.2025.101163)
Supplement: Supplementary file 1 — Supplementary material: Original questionnaire (Fig. S1) and results of the ordinal logistic regression analyses (Table S1). [file mmc1.pdf]

Supplementary Information

Health literacy, attitudes and preventive practices concerning mosquitoes and mosquito-borne infections – a questionnaire survey in a German community

Lukas Eicher[1], Andrea Weber[1], Julia Tobias[2], Andrea Verbitskii[2], Michael Leitzmann[1], Benedikt MJ Lampl[1,2]

[1]Department of Epidemiology and Preventive Medicine, University of Regensburg, Regensburg, Germany
[2]Division of Infection Control and Prevention, Regensburg Department of Public Health, Regensburg, Germany

Correspondence:
Benedikt M J Lampl, MD
Department of Epidemiology and Preventive Medicine
Franz-Josef-Strauß-Allee 11
93053 Regensburg, Germany
Phone +49-941 4009-523
Email: Benedikt.Lampl@klinik.uni-regensburg.de

This PDF file includes Figure S1 and Table S1.

Figure S1: Questionnaire (original German version)

Fragebogen

Gesundheitskompetenz in puncto stechmücken-übertragener Infektionen und Mückenschutz (GeKoMü-Studie)

UKR Universitätsklinikum Regensburg

Landratsamt Regensburg Gesundheitsamt Regensburg

Persönliche Daten

(1)	**Bitte wählen Sie Ihr *Geschlecht* und Ihr *Alter* aus.**<br>☐männlich<br>☐weiblich<br>☐keine Angabe	☐unter 20 Jahre<br>☐20 - 40 Jahre<br>☐über 40 - 60 Jahre<br>☐über 60 Jahre

(2)	**Bitte wählen Sie Ihren *höchsten Bildungsabschluss* aus.**<br>(*Mehrfachauswahl möglich*)<br>☐Kein Abschluss<br>☐Abschluss im medizinischen / biologischen Bereich<br>☐Mittelschulabschluss (Hauptschul-, Volksschulabschluss)<br>☐Realschulabschluss (Mittlere Reife)<br>☐Allgemeine oder fachgebundene Hochschulreife / Abitur<br>☐Berufsausbildung<br>☐ Abschluss an einer (Fach-) Hochschule / Universität<br>☐ Anderer Abschluss

Reiseerfahrung

(3)	**Ich stamme gebürtig aus Deutschland.**	☐ja<br>☐nein, sondern aus ________________ .

(4)	**Ich bin bereits in die (Sub-) Tropen gereist (z.B. Brasilien, Thailand, südliches Afrika …).**	☐ja<br>☐nein

Fragebogen

Gesundheitskompetenz in puncto stechmücken-übertragener Infektionen und Mückenschutz (GeKoMü-Studie)

Universitätsklinikum Regensburg

Landratsamt Regensburg Gesundheitsamt Regensburg

Bitte beantworten Sie die folgenden Fragen, wenn sie bei (4) *Ja* angekreuzt haben.

**(5)**	**Vor meiner Reise wurde ich reisemedizinisch beraten (z.B. beim Hausarzt).**	☐ja<br>☐nein

**(6)**	**Bei meiner Beratung wurde ich über von Mücken übertragene Krankheiten und Mückenschutz aufgeklärt.**	☐ja<br>☐nein

Wissensstand zu Mücken und Schutzmaßnahmen

**(7)**	**Wo brüten Mücken bevorzugt? In…**<br>(*Mehrfachauswahl möglich*)<br><br>☐stehenden Gewässern (Seen, Teiche)<br>☐offenen Wasserquellen (Regentonnen)<br>☐feuchter Erde (Blumentöpfe)<br>☐trockener Erde (Ackerboden)<br>☐künstlichen Wasserstellen (Plastikmüll)<br>☐Wäldern (auf Blättern)<br><br>☐Keine dieser Angaben ist richtig.

**(8)**	**Von welcher Mückenart haben Sie bereits gehört?**<br>(*Mehrfachauswahl möglich*)<br><br>☐Gelbfieber-Mücke (Aedes-Mücke)<br>☐Malaria-Mücke (Anopheles-Mücke)<br>☐Gemeine Stechmücke (Culex-Mücke)<br><br>☐Ich habe bisher von keiner dieser Mückenarten gehört.

Fragebogen

Gesundheitskompetenz in puncto stechmücken-übertragener Infektionen und Mückenschutz (GeKoMü-Studie)

Landratsamt Regensburg Gesundheitsamt Regensburg

UKR Universitätsklinikum Regensburg

**(9)**	**Welche der folgenden Krankheiten wird durch Stechmücken übertragen?**<br><br>(*Mehrfachauswahl möglich*)<br><br>☐Malaria<br>☐Dengue-Fieber<br>☐Gelbfieber<br>☐Zika-Virus<br>☐West-Nil-Virus<br>☐Chikungunya-Fieber<br>☐ HIV<br><br>☐Keine dieser Krankheiten wird durch Stechmücken übertragen.

Einstellung gegenüber Mückenschutz

Die nachfolgenden Fragen beziehen sich jeweils auf **Ihre Meinung** zum Thema Mückenschutz in Deutschland. Bitte kreuzen Sie bei den folgenden Fragen an, was *am meisten* auf Sie zutrifft.

(10) Aktuell sehe ich eine Gefahr durch stechmückenübertragene Infektionen in Deutschland.

**1**<br>stimme vollkommen zu	**2**<br>stimme zu	**3**<br>weder noch	**4**<br>stimme nicht zu	**5**<br>stimme überhaupt nicht zu
☐	☐	☐	☐	☐

(11) Ich glaube, dass der Klimawandel in Zukunft Auswirkungen auf unsere Gesundheit haben wird (z.B. Infektionskrankheiten).

**1**<br>stimme vollkommen zu	**2**<br>stimme zu	**3**<br>weder noch	**4**<br>stimme nicht zu	**5**<br>stimme überhaupt nicht zu
☐	☐	☐	☐	☐

(12) Durch den Klimawandel werden immer mehr nicht heimische Mücken in Deutschland vorkommen.

**1**<br>stimme vollkommen zu	**2**<br>stimme zu	**3**<br>weder noch	**4**<br>stimme nicht zu	**5**<br>stimme überhaupt nicht zu
☐	☐	☐	☐	☐

Fragebogen

Gesundheitskompetenz in puncto stechmücken-übertragener Infektionen und Mückenschutz (GeKoMü-Studie)

Landratsamt Regensburg Gesundheitsamt Regensburg

UKR Universitätsklinikum Regensburg

(13) Weil Mücken Krankheiten übertragen können, werden Tropenkrankheiten in Deutschland eine größere Rolle spielen.				
**1** stimme vollkommen zu	**2** stimme zu	**3** weder noch	**4** stimme nicht zu	**5** stimme überhaupt nicht zu
☐	☐	☐	☐	☐

(14) Weil Mücken mögliche Krankheitsüberträger sind, sollte der Staat den Umgang mit ihnen über Gesetze regeln.				
**1** stimme vollkommen zu	**2** stimme zu	**3** weder noch	**4** stimme nicht zu	**5** stimme überhaupt nicht zu
☐	☐	☐	☐	☐

(15) Ich finde es sinnvoll, Mücken genetisch zu verändern, um deren Ausbreitung zu verhindern.				
**1** stimme vollkommen zu	**2** stimme zu	**3** weder noch	**4** stimme nicht zu	**5** stimme überhaupt nicht zu
☐	☐	☐	☐	☐

Handlungsbereitschaft

Die nachfolgenden Fragen bewerten Ihre **Bereitschaft, selbst gegen die Ausbreitung von Mücken zu handeln**. Bitte kreuzen Sie bei den folgenden Fragen an, was *am meisten* auf Sie zutrifft.

(16) Ich schütze mich jetzt schon bewusst vor Mücken.				
**1** stimme vollkommen zu	**2** stimme zu	**3** weder noch	**4** stimme nicht zu	**5** stimme überhaupt nicht zu
☐	☐	☐	☐	☐

Fragebogen

Gesundheitskompetenz in puncto stechmücken-übertragener Infektionen und Mückenschutz (GeKoMü-Studie)

Universitätsklinikum Regensburg

Landratsamt Regensburg Gesundheitsamt Regensburg

**(17)**	**Welches dieser Mittel nutzen Sie regelmäßig im Alltag zum Schutz vor Mücken (während der Mückensaison)?**<br><br>(*Mehrfachauswahl möglich*)<br><br>☐Lange Kleidung<br>☐Insektenspray<br>☐Moskitonetze oder Mückengitter<br>☐Ventilator oder Klimaanlage<br>☐ Mückenfallen (mit Lockstoffen, z.B. CO_2)<br>☐Elektrogeräte (z.B. akustische Signale, UV-Licht)<br>☐Duftstoffe für die Umgebung (z.B. Zitronella-Kerzen)<br>☐Insektengift (z.B. Bti-Toxin Pellets / Tabletten)<br>☐Knoblauch essen<br><br>☐Ich verwende keines dieser Mittel.

(18) Wenn Mücken in Zukunft ein größeres Problem für die Gesundheit darstellen, werde ich mich bewusst gegen sie schützen.

**1**<br>stimme vollkommen zu	**2**<br>stimme zu	**3**<br>weder noch	**4**<br>stimme nicht zu	**5**<br>stimme überhaupt nicht zu
☐	☐	☐	☐	☐

(19) Ich entferne bewusst mögliche Brutplätze von Mücken aus meiner Umgebung (stehende Wasserquellen wie Regentonnen, Blumentöpfe, Plastikbehälter usw.).

**1**<br>stimme vollkommen zu	**2**<br>stimme zu	**3**<br>weder noch	**4**<br>stimme nicht zu	**5**<br>stimme überhaupt nicht zu
☐	☐	☐	☐	☐

Vielen Dank für Ihre Teilnahme!

Table S1: Results of the ordinal logistic regression analyses.

	**Dependent variable**							
	**Score question (7):** *"Where do mosquitoes prefer to breed?"*		**Score question (8):** *"Which type of mosquito have you already heard of?"*		**Score question (9):** *"Which of the following diseases is transmitted by mosquitoes?"*		**Overall score in all knowledge questions** (Questions (7)-(9))	
**Independent variable**	**p**	**β**	**p**	**β**	**p**	**β**	**p**	**β**
**Gender** [1]	0.0416	-0.3371	0.073	-0.2957	0.00677	-0.4295	0.0110	-0.4003
**Age** [2]	0.00358	0.5705	0.00186	0.6108	0.000131	0.7338	0.00000595	0.88752
**Education** [3]	0.130	-0.2420	0.00506	-0.4484	0.000000252	-0.8041	0.0000129	-0.6665
**Experienced in traveling to the (sub-)tropics** [4]	0.112	0.2680	0.0363	0.3531	0.00073	0.5446	0.000717	0.5360
**Received pre-travel health advice** [4]	0.157	0.4784	0.000556	1.21257	0.00225	1.0149	0.0000103	1.5193
**Received information about mosquito-borne diseases and mosquito protection** [4]	0.0291	0.7001	0.0122	0.81895	0.00427	0.8951	0.000104	1.2113

[1] Adjustment for age and education; regression coefficient β refers to female gender.

[2] Adjustment for gender and education; regression coefficient β refers to participants aged > 40 years.

[3] Adjustment for gender and age; regression coefficient β refers to participants without a college or university degree.

[4] Adjustment for gender, age and education; regression coefficient β refers to participants who answered "yes".

Table S1 (continued)

	Dependent variable					
	**(10):** *"There is currently a risk of mosquito-borne infections in Germany."*		**(11):** *"Climate change will have an impact on our health in the future."*		**(12):** *"Due to climate change, more and more non-native mosquitoes will be found in Germany."*	
**Independent variable**	**p**	**β**	**p**	**β**	**p**	**β**
**Gender [1]**	0.514	-0.1031	0.0533	-0.3264	0.0662	-0.3089
**Age [2]**	0.0000126	-0.8509	0.202	0.2546	0.0891	0.3402
**Education [3]**	0.166	-0.2135	0.00203	0.5072	0.000176	0.6158
**Experienced in traveling to the (sub-) tropics [4]**	0.444	0.1243	0.835	-0.03625	0.552	-0.1033
**Received pre-travel health advice [4]**	0.242	-0.3705	0.0781	-0.6222	0.0281	-0.7412
**Received information about mosquito-borne diseases and mosquito protection [4]**	0.775	-0.08726	0.0488	-0.6600	0.230	-0.3906
**Question (7): score [5]**	0.300	0.23918	0.452	0.1840	0.848	0.0465
**Question (8): score [6]**	0.848	0.03885	0.00000458	1.0019	0.00000000255	1.3202
**Question (9): score [7]**	0.0065	0.4734	0.0137	0.4571	0.0000166	0.8022
**Questions (7)-(9): overall score [8]**	0.00213	0.4990	0.0000475	0.7147	0.00000614	0.7865

[1] Adjustment for age and education; regression coefficient β refers to female gender.
[2] Adjustment for gender and education; regression coefficient β refers to participants aged > 40 years.
[3] Adjustment for gender and age; regression coefficient β refers to participants without a college or university degree.
[4] Adjustment for gender, age and education; regression coefficient β refers to participants who answered "yes".
[5] Adjustment for gender, age and education; regression coefficient β refers to participants with a score of 2 or lower.
[6] Adjustment for gender, age and education; regression coefficient β refers to participants with a score of 1 or lower.
[7] Adjustment for gender, age and education; regression coefficient β refers to participants with a score of 3 or lower.
[8] Adjustment for gender, age and education; regression coefficient β refers to participants with a score of 6 or lower.

Table S1 (continued)

	Dependent variable					
	**(13):** *"Since mosquitoes can transmit diseases, tropical diseases will play a more important role in Germany."*		**(14):** *"Since mosquitoes are potential transmitters of diseases, the government should create laws to regulate their treatment."*		**(15):** *"It is useful to modify mosquitoes' genes to prevent them from spreading."*	
**Independent variable**	**p**	**β**	**p**	**β**	**p**	**β**
**Gender [1]**	0.189	-0.2269	0.0701	0.2926	0.149	0.22940
**Age [2]**	0.557	0.1213	0.0131	0.4904	0.005798	0.52275
**Education [3]**	0.0401	0.3454	0.213	0.1942	0.748	0.04925
**Experienced in traveling to the (sub-) tropics [4]**	0.861	-0.03106	0.846	0.0320	0.582	-0.08852
**Received pre-travel health advice [4]**	0.0404	-0.6957	0.130	-0.4829	0.721	0.11239
**Received information about mosquito-borne diseases and mosquito protection [4]**	0.143	-0.4764	0.266	-0.3344	0.824	0.06661
**Question (7): score [5]**	0.670	0.1080	0.632	0.1132	0.211	-0.29653
**Question (8): score [6]**	0.00086	0.7461	0.0199	0.4754	0.279	0.21715
**Question (9): score [7]**	0.000041	0.7756	0.200	0.2256	0.229	0.20893
**Questions (7)-(9): overall score [8]**	0.0000156	0.7761	0.133	0.2448	0.766	-0.04759

[1] Adjustment for age and education; regression coefficient β refers to female gender.

[2] Adjustment for gender and education; regression coefficient β refers to participants aged > 40 years.

[3] Adjustment for gender and age; regression coefficient β refers to participants without a college or university degree.

[4] Adjustment for gender, age and education; regression coefficient β refers to participants who answered "yes".

[5] Adjustment for gender, age and education; regression coefficient β refers to participants with a score of 2 or lower.

[6] Adjustment for gender, age and education; regression coefficient β refers to participants with a score of 1 or lower.

[7] Adjustment for gender, age and education; regression coefficient β refers to participants with a score of 3 or lower.

[8] Adjustment for gender, age and education; regression coefficient β refers to participants with a score of 6 or lower.

Table S1 (continued)

	Dependent variable					
	**(16):** *"I already consciously protect myself from mosquitoes."*		**(18):** *"If mosquitoes become a bigger health problem in the future, I will consciously protect myself against them."*		**(19):** *"I consciously remove potential breeding sites for mosquitoes from my surroundings."*	
**Independent variable**	**p**	**β**	**p**	**β**	**p**	**β**
**Gender [1]**	0.00244	-0.4929	0.05978	-0.3299	0.00272	-0.4696
**Age [2]**	0.000223	-0.7318	0.216	0.2613	0.000201	-0.6829
**Education [3]**	0.395	-0.1351	0.617	0.0855	0.444	0.1173
**Experienced in traveling to the (sub-) tropics [4]**	0.0984	-0.2757	0.255	-0.20496	0.833	0.03367
**Received pre-travel health advice [4]**	0.0138	-0.8010	0.0800	-0.6403	0.00111	-1.04921
**Received information about mosquito-borne diseases and mosquito protection [4]**	0.00068	-1.0854	0.068	-0.6209	0.07499	-0.5460
**Question (7): score [5]**	0.763	-0.07159	0.345	0.24036	0.473	0.16970
**Question (8): score [6]**	0.391	0.1780	0.00132	0.737745	0.273	0.22560
**Question (9): score [7]**	0.0655	0.3271	0.007859	0.51033	0.275	0.1862
**Questions (7)-(9): overall score [8]**	0.03067	0.3583	0.06689	0.32986	0.004889	0.45293

[1] Adjustment for age and education; regression coefficient β refers to female gender.

[2] Adjustment for gender and education; regression coefficient β refers to participants aged > 40 years.

[3] Adjustment for gender and age; regression coefficient β refers to participants without a college or university degree.

[4] Adjustment for gender, age and education; regression coefficient β refers to participants who answered "yes".

[5] Adjustment for gender, age and education; regression coefficient β refers to participants with a score of 2 or lower.

[6] Adjustment for gender, age and education; regression coefficient β refers to participants with a score of 1 or lower.

[7] Adjustment for gender, age and education; regression coefficient β refers to participants with a score of 3 or lower.

[8] Adjustment for gender, age and education; regression coefficient β refers to participants with a score of 6 or lower.
